# Assessment of the Transfer of Trace Metals to Spontaneous Plants on Abandoned Pyrrhotite Mine: Potential Application for Phytostabilization of Phosphate Wastes

**DOI:** 10.3390/plants11020179

**Published:** 2022-01-11

**Authors:** Meryem El Berkaoui, Mariam El Adnani, Rachid Hakkou, Ahmed Ouhammou, Najib Bendaou, Abdelaziz Smouni

**Affiliations:** 1 Laboratory of Plant Physiology and Biotechnology, Laboratoire Mixte International—LMI AMIR, Research Center on Plant and Microbial Biotechnology, Biodiversity and Environment, Faculty of Sciences, Mohammed V University in Rabat, Rabat 10001, Morocco; n.bendaou@um5r.ac.ma; 2 Mining and Environmental Engineering Laboratory, National School of Mines of Rabat, l Av. Hadj Ahmed Cherkaoui, BP 753, Rabat 53000, Morocco; eladnani@enim.ac.ma; 3 IMED-Lab, Faculty of Sciences and Technology, Cadi Ayyad University, BP 549, Marrakech 40000, Morocco; r.hakkou@uca.ma; 4 Mining Environment & Circular Economy (EMEC) Program, Mohammed VI Polytechnic University (UM6P), Ben Guerir 43150, Morocco; 5 Laboratory of Microbial Biotechnology, Agrosciences and Environment, Faculty of Sciences-Semlalia, Cadi Ayyad University, BP 2390, Marrakech 40000, Morocco; ouhammou@uca.ac.ma

**Keywords:** phytostabilization, phytoremediation, metals, accumulation, plants, soils, mine, phosphate

## Abstract

The abandoned Kettara pyrrhotite mine (Marrakech region, Morocco) is a real source of acid mine drainage (AMD) and heavy metal pollution from previous mining operations—which has spread, particularly because of wind erosion. A store-and-release cover system made of phosphate wastes was built on the site for preventing AMD. To ensure the integrity of this cover and its durability, it is desirable to revegetate it (phytostabilization) with plants adapted to the edaphoclimatic conditions of the region. In this paper, a study was carried out on the spontaneous vegetation around the phosphate cover in order to consider the selection of plants to promote the stabilization of the Kettara mine tailings pond. Nine species of native plants with their rhizospheric soils growing in agricultural soils and tailings from the Kettara mine were collected, and metals (As, Cd, Co, Cu, Pb, Zn, Ni, Cr) were analyzed. The soil analysis showed that the tailings contained high concentrations of Cu (177.64 mg/kg) and Pb (116.80 mg/kg) and that the agricultural soil contained high concentrations of As (25.07 mg/kg) and Cu (251.96 mg/kg) exceeding the toxicity level (Cu > 100 mg/kg, Pb > 100 mg/kg, As > 20 mg/kg). The plant analysis showed low trace metal accumulation in *Scolymus hispanicus*, *Festuca ovina, Cleome brachycarpa, Carlina involucrata* and *Peganum harmala*. These species had a bioconcentration factor (BCF) greater than 1 and a translocation factor (TF) less than 1, demonstrating a high tolerance to trace metals. Therefore, they are good candidates for use in the phytoremediation of the Kettara mine tailings. These species could also potentially be used for the phytostabilization of the phosphate waste cover of the Kettara mine, thus completing the rehabilitation process of this area.

## 1. Introduction

Among the main pollutants generated by mining activities, metals and metalloids are of particular concern. These elements, which are naturally non-biodegradable, exhibit high ecotoxicity and cause major damage to environment. Thus, it is essential to implement long-term solutions to limit their pollution potential [1]. The 2030 Agenda for Sustainable Development, which includes 17 Sustainable Development Goals (SDGs), also refers to this issue. By 2030, SDG target 15.3 aims to restore degraded lands and soils, including abandoned mine sites. In this context, remediation strategies have been developed to prevent the dispersion of pollutants, stabilize surfaces, prevent erosion, and recover abandoned mine sites [2,3].

Plants play an important role in removing pollutants and hazardous substances from the environment, and their use is effective in restoring soil fertility. The use of plants in abandoned mining areas to prevent erosion and for plant stabilization is a phytoremediation technique that aims to immobilize and stabilize pollutants in contaminated soils [4,5]. Phytostabilization is one of the phytoremediation techniques that allow plant cover to be established on the surface of polluted soils to reduce not only the bioavailability and mobility of contaminants but also their availability and thus prevent the transfer of elements into the food chain [6,7,8,9].

This type of phytoremediation technique must not only be based on the accumulation of heavy metals but also on whether they adapt to the edaphoclimatic conditions of the region. The solution is to select dominant plants that grow spontaneously in the region with a capacity for phytoremediation. The selection of plant species from contaminated areas is an important condition for a future systemic phytoremediation because it allows one to target plants capable of tolerating a high concentration of metals [10,11,12,13,14,15,16,17].

The abandoned Kettara pyrrhotite mine, located 30 km northwest of Marrakech (Morocco), is a district rich in metals [18,19]. The pollution problem of this mine site is mainly due to the phenomenon of acid mine drainage (AMD). AMD is produced by sulfurous minerals present in the ore, pyrite and pyrrhotite, exposed to water and the atmospheric air. This acidity, combined with the presence of metals and metalloids, can seriously affect the ecosystems that receive the contaminated effluents. In order to reduce AMD from this mine site, previous work resulted in the construction of a hydrogeological (store-and-release) cover system based on phosphate waste for reducing water infiltration, in order to rehabilitate this former mining site. To finalize the rehabilitation plan, a vegetation study must be carried out, on the one hand, to develop an effective and inexpensive way of stabilizing the cover and, on the other, to ensure its durability and therefore the reintegration of the soil [20,21,22,23,24,25].

In this study, the focus has been put on the dominant native and spontaneous vegetation of the Kettara mine site. This vegetation has the ability to tolerate high concentrations of metals, as well as to adapt to the climatic conditions of the contaminated site. In fact, one should select plants capable of stabilizing the Kettara mine site with a view of developing the phytoremediation technique of the contaminated zone. In this article, the authors sought to assess the mobility and phytoavailability of metals and metalloids both by chemical extraction procedures and by the analysis of metal transfer in shoots and roots of plants growing in the Kettara mine site area. This characterization aimed to identify plant species that can be used in plant phytostabilization programs on mining sites.

## 2. Materials and Methods

### 2.1. Study Area and Sampling

**Figure 1:**
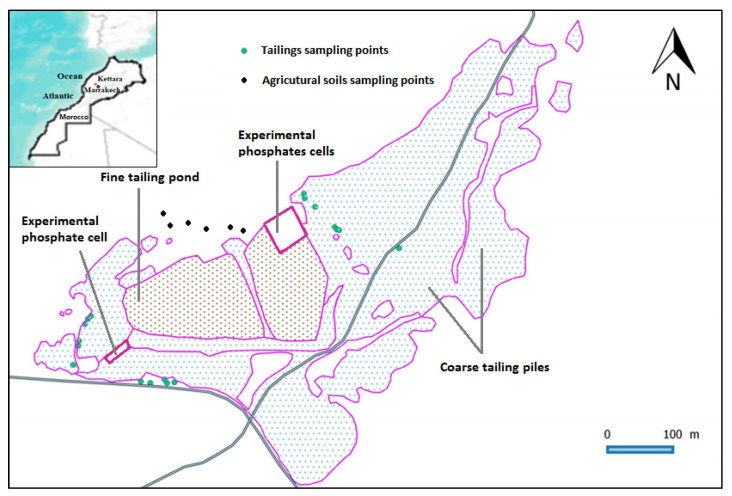
Geographical location of Kettara mine site and location of sampling area on tailings and agricultural soils.

The Kettara mining village of about 1169 inhabitants is located 30 km northwest of Marrakech (Morocco) (Figure 1). An underground pyrrhotite mine was operated there from 1964 to 1981. The climate of the region is semi-arid. The annual precipitation is low (≤250 mm) and irregular. The relative humidity and the temperature are, on average, from 73% and 12 °C in January to 33% and 29 °C in July, respectively [26].

Nine plant species were identified as dominant and growing spontaneously in two zones of the Kettara mine site: the tailings zone and the agricultural soil zone representing the surroundings of the phosphate waste (Figure 1). Individuals of each species, with their aerial and root parts and with their rhizospheric soils, were sampled randomly during the months of May and June in order to ensure a maximum of drought-resistant species in this study region. The species selection was based on the dominance of plants that grow spontaneously in the study areas due to their tolerance to metals and their adaptation to the climatic and edaphic conditions of the region. There was a total of 9 species belonging to 6 families according to the nomenclature of the “Practical Flora of Morocco” [27,28], of which 3 belong to Asteraceae and 2 belong to Poaceae.

The species in the agricultural soils were *Festuca ovina* (Poaceae), *Colocynthis vulgaris* (Cucurbitaceae), *Glaucium corniculata* (Papaveraceae), *Peganum harmala* (Asteraceae), *Cleome brachycarpa* (Capparaceae) and *Stipa retorta* (Poaceae). The species in the tailings were *Festuca ovina* (Poaceae), *Carlina involucrata* (Asteraceae), *Salvia aegyptiaca* (Lamiaceae), *Colocynthis vulgaris* (Cucurbitaceae), *Scolymus hispanicus* (Asteraceae), *Peganum harmala* (Asteraceae) and *Cleome bracharaceae* (Capparaceae). Four common plant species (*Festuca ovina, Colocynthis vulgaris, Peganum harmala* and *Cleome brachycarpa*) were found in the two areas.

Soil sampling was carried out at a depth of 10 cm and over an area of 0.3 m^2^. The samples were homogenized and sieved to less than 2 mm [29]. The geographic coordinates of the sampling points were obtained with a global positioning system (GPS) with an accuracy of ±5 m and a Lambert Nord Morocco cartographic projection.

### 2.2. Plant and Soil Samples Analysis

Plant samples were washed with tap water and rinsed with distilled water. Then, the samples were dried for 3 consecutive days in the oven at 80 °C. The dried tissues were ground and mineralized according to the acid hydrolysis protocol described by [30].

The samples from the agricultural soil rhizosphere soil and the tailings were dried at 80 °C and then ground to a particle size of less than 180 mm. Then, 2 mL of concentrated HNO_3_ was added to each 100 mg of soil and brought to dryness at 110 °C. After maintaining the solution for 15 h at 140 °C, 3 mL of concentrated hydrofluoric acid was added to the solution; subsequently the solution was cooled to 110 °C, and 2 mL of concentrated HNO_3_ was added. This operation was repeated three times to obtain a total of 6 mL of HNO_3_. Finally, 25 mL of 2 M HCl was added to the dry extracts before being analyzed. The total metal concentration of nickel (Ni), cobalt (Co), chromium (Cr), zinc (Zn), copper (Cu), lead (Pb), cadmium (Cd), and arsenic (As) was determined by Inductively Coupled Plasma Atomic Emission Spectrometry (ICP-AES) [29].

The mobile and mobilizable metal fractions were estimated separately using 50 millimolar (mM) of EDTA and 10 mM of CaCl_2_ at neutral pH, respectively. For each soil sample, 2 g was suspended in 50 mM EDTA for the mobilizable and 20 mL of CaCl_2_ for the mobile and stirred for 2 h; then, centrifugation for 12 min at 8000 rpm. Finally, the concentration of mobile and mobilizable metals was determined by ICP-AES on the supernatants and at a wavelength of 220.353 nm [27]. For the pH measurement, 10 g of soil was suspended in 50 mL of distilled water with stirring for 1 hour using a magnetic stirrer and then decanted for 30 min. The pH was also determined on the supernatant [31].

### 2.3. Bioconcentration and Translocation Factors

The bioconcentration factor (BCF) reflects the ability of a plant to accumulate a metal in its tissues from the soil, and it is calculated as the ratio of the concentration of metal in plant tissues to the concentration of total metal in the soil, as given by Equation (1) [32]. A BCF less than 1 indicates a greater accumulation of metals in the root system, while a BCF greater than 1 indicates a higher concentration in the tissues of the plant.

The translocation factor (TF) indicates the translocation of the metal from the root to the shoot of plants and is calculated as the ratio of the concentration of metal in aerial tissues to the concentration of metal in the root of the plant, as given by Equation (2) [33].

(1) $$\mathrm{BCF}=\frac{C-\mathrm{plant}\;\mathrm{tissue}\;}{C-\mathrm{soil}}\;$$

(2) $$\mathrm{TF}=\frac{C-\mathrm{shoot}}{C-\mathrm{root}}$$

Plant species with TF greater than BCF could be used in phytoextraction, while plant species with BCF greater than 1 and low TF could be considered as candidates for phytostabilization.

### 2.4. Statistical Analysis

The statistical analysis of metal content in the soils was performed using the SPSS software package version 2. It was based on analysis of variance (ANOVA) test. The mean was calculated with standard deviation (SD) using the Student–Newman–Keuls grouping test. The statistical significance was declared when *p* ≤ 0.05.

## 3. Results and Discussion

### 3.1. Total Content and Availability of Metals and Metalloids in the Soils

The total content of eight metals and the relative standard deviations in the tailings area and agricultural soil area are reported in Table 1. Cu was significantly the higher total metal content (*p* < 0.05) in the two zones, and it was 2 times higher than the toxicity levels with an average of 177.64 mg/kg in the tailings and 251.96 mg/kg in the agricultural soil. Pb was significantly the higher total metal content (*p* < 0.05) in the tailings with an average of 116.80 mg/kg exceeded Pb toxicity levels. As recorded the significantly higher content levels (*p* < 0.05) than toxicity levels in the agricultural soil were recorded with an average of 25.07 mg/kg [34].

Regarding the pH, the rhizospheric soil samples from two different locations revealed that the pH of soils varied within the range 6.87–7.34. The surrounding area of the mine site was slightly alkaline, with medium acidity (6.87) in the tailings and a neutral pH of the agricultural soil (7.34), which is appropriate for normal growth and development of vegetation.

This study is in agreement with that of Khalil et al. (2013) [22] on the soil contamination of the pyrrhotite mine in Morocco. They also found high total concentrations of As and Cu in the soils. According to [22], the Cu was released mainly from the mine tailings located within the northern side of the study area, which was associated with mine waste from the exploitation of pyrite and pyrrhotite. The origin of Cu in the mine tailings was chalcopyrite [18], which was present in the mineral paragenesis of Kettara [35]. For As, according to Khalil et al. (2013), the spatial distribution of As in the Kettara soil showed that the origin of this metal is anthropogenic in the deposited mine tailings. This mineral spread out in the study area mainly by surface water and wind, following a leaching of mine tailings by AMD with the weathering process. About Pb, this element was leached from the galena contained in the mine tailings by AMD generation. Galena was left in the mine tailings during the Kettara mining exploitation.

In addition, these results are in agreement with a study done by Wu et al. (2021) [17] on the accumulation of metals in native plants of a pyrite mine in China. Similar to the results of the present study, they found high total Cu concentrations in tailings and far exceeded the Class-II standard for China’s soil and environmental quality.

In both zones, the mobile fraction (CaCl_2_) of the metals and metalloids (As, Cd, Co, Cr, Cu, Ni, Pb, Zn) was close to zero and practically insoluble (Table 1). This indicates that the leaching under natural conditions is very low. These results are probably due to the pH substrate nature that was neutral to slightly alkaline. In both study areas, the extraction with EDTA does not allow the release of As (0.15 mg/kg in the tailings, and 0.26 mg/kg in the agricultural soil), Cd (0.2 in the tailings, and 0.02 in the agricultural soil) and Co (0.08 in the tailings, and 0.53 in the agricultural soil). However, it generates an amount of metals (Cu, Pb and Zn) suggesting that they are potentially available. This fraction containing acceptable trace elements can be released into the soil solutions. In fact, the metal potential availability can be expressed as follows: Cu > Pb > Zn > As > Co > Cd.

**Figure 2:**
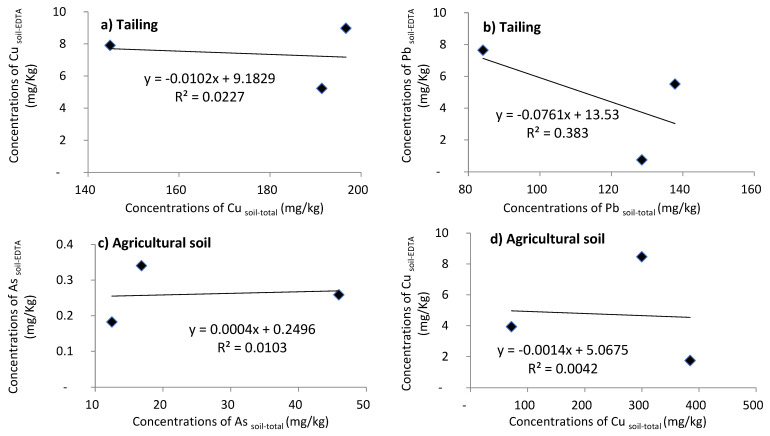
Correlation between total (**a**) Cu and (**b**) Pb content and its bioavailable fractions in tailings, and the correlation between total (**c**) As and (**d**) Cu content and its bioavailable fractions in agricultural soil.

The toxic impact of metals on ecosystems and human health is caused by content of their bioavailable forms. To identify factors affecting the content of bioavailable Cu, Pb and As, a correlation analysis was performed, and the results are shown in Figure 2.

For Cu, the results of the total Cu content in the tailings presented 2% (R^2^ = 0.02) of bioavailable Cu, and in the agricultural soil, the total Cu content presented 0.4% (R^2^ = 0.004) of bioavailable Cu, which means a very low correlation between total Cu content and its bioavailable fractions. The same was recorded for As in the agricultural soil whose total As content presented 1% (R^2^ = 0.01) of bioavailable As. In contrast, the Pb showed in the tailings an average correlation with a total Pb content presenting 38% (R^2^ = 0.383) of bioavailable Pb.

The low correlation is probably conditioned by the ability of Cu and As to be incorporated and bound to soil organic matter. They are not easily leached into the agricultural soil and the tailings and become biologically unavailable [36].

### 3.2. Metal Accumulation in Plants

**Figure 3:**
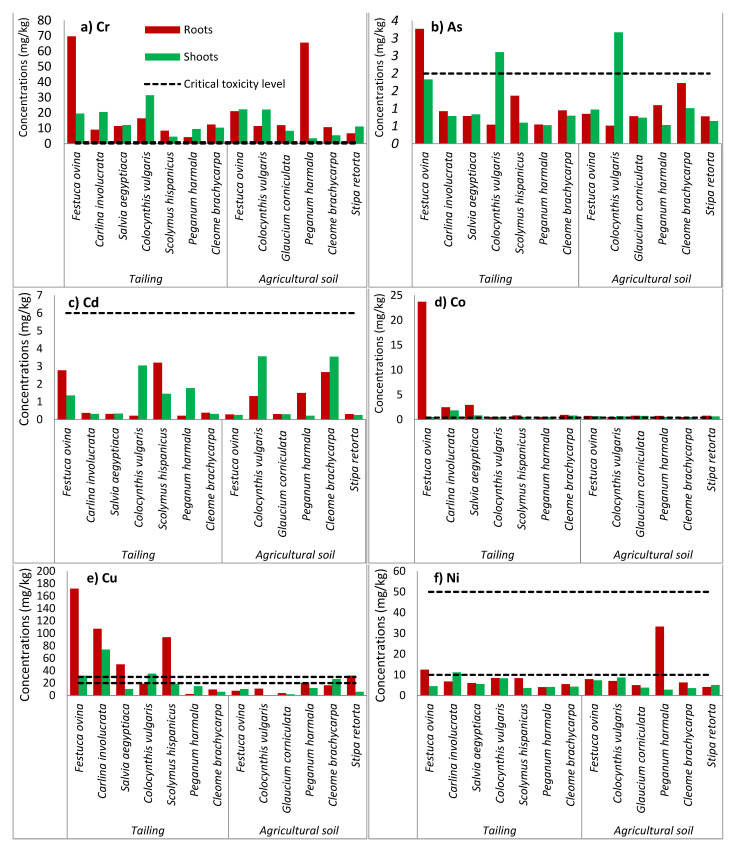

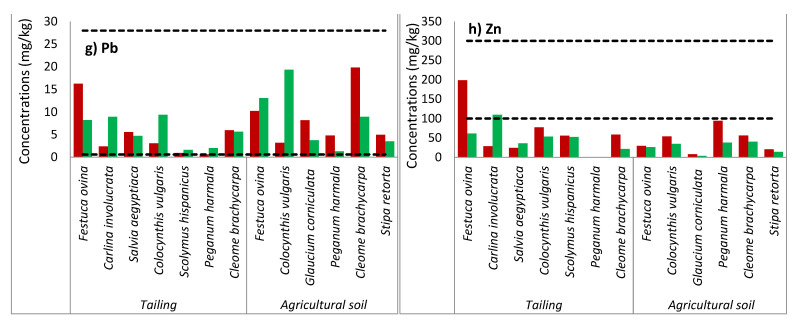
Concentration of (**a**) Cr, (**b**) As, (**c**) Cd, (**d**) Co, (**e**) Cu, (**f**) Ni, (**g**) Pb, and (**h**) Zn in shoots and roots (mg/kg dry weight) of species growing in tailings and in agricultural soil at Kettara abandoned mine.

The metals and metalloids in the shoots and roots of the dominant plants collected in the two areas are shown in Figure 3.

The metals Co, Cr and Pb showed a high concentration and exceeded the level of toxicity [34] in the shoots and roots of all plants in the two study areas (Figure 3).

Depending on the different metal tolerance of a given plant, phytotoxic ranges of examined metals and metalloids were reported by [34,37], which were <2–80 mg/kg for As, 6–10 mg/kg for Cd, 0.4–several mg/kg for Co, 0.2–1 mg/kg for Cr, 20–30 mg/kg for Cu, 10–50 mg/kg for Ni, 0.6–28 mg/kg for Pb and 100–300 mg/kg for Zn.

The highest content of Cr was recorded in the *Festuca ovina* tissues of the two zones: with a value of 69.69 times the level of toxicity for the roots in the tailings, and with a value of 22.27 times the toxicity level for the aerial part in the agricultural soil. A high level of Cr was also recorded in the roots of *Peganum harmala* (65.4 times, agricultural soil) and in the shoots of *Colocynthis vulgaris* (31.42 times, tailings).

The highest concentration of Co was found in the tailings area, in the roots of *Festuca Ovina* (59.17 times the toxicity level of Co) and in the shoots of *Carlina involucrate* (4.6 times the level of Co toxicity). In addition, the concentration of Co in the roots is much higher than in the shoots. The ratio of the Co concentration in roots to shoots may indicate that a protective mechanism has been initiated by plants highly exposed to Co to reduce the uptake of metals [38].

The highest content of Pb in shoots was in *Colocynthis vulgaris* in both areas (1.63 times in the tailings, and 32.22 times in the agricultural soil), in roots of *Festuca ovina* (27.07 times in the tailings), and in *Cleome brachycarpa* (33.02 times in the agricultural soil).

The results also showed high Cu contents in the different parts of *Festuca ovina* (1.59 times in the shoot, 8.58 times in the roots) and in *Carlina involucrata* (30.70 times in the shoots, 5.36 times in the roots) pushed into the tailings.

The concentrations of As, Cd, Cu, Ni and Zn in the plant tissues showed values below their level of toxicity in the shoots and roots. This is probably due to the low bioavailable content in the soil, which resulted in a reduced transfer of metals to these plants with physiological peculiarities of the plants [36].

In this study, the results indicated no hyperaccumulation since none of the concentrations of As, Cd, Co, Cr, Cu, Ni, Pb and Zn in the plant tissues reached critical levels, and they accumulated less >1000 mg/kg for Pb, >100 mg/kg for Cd, >1000 mg/kg for As, >1000 mg/kg for Cu, >1000 mg/kg for Cr, >1000 mg/kg for Co, >1000 mg/kg for Ni, and >10,000 mg/kg for Zn, respectively, as reported by [34,37].

One can conclude that the sampled plant species can accumulate metals and metalloids (As, Cd, Co, Cr, Cu, Ni, Pb and Zn) in their shoots and roots. These results could indicate that plant species growing in the abandoned Kettara mine tolerate these metals.

### 3.3. Phytostabilisation Potential of Plant Species

Phytostabilization of mine tailings needs the use of metal-tolerant plants for the immobilization of metals and metalloids in the tailings’ substrate [39]. The bioconcentration factor is a significant indicator of plant species for phytostabilization suitability [36].

In this study, none of the studied plants can be classified as hyperaccumulators [34,37,40]. However, the ability of these plants to tolerate and accumulate metals will be useful for phytostabilization. Both bioconcentration factor (BCF) and translocation factor (TF) can be used to estimate a plant’s potential for a phytostabilization purpose. BCF is defined as the ratio of metal concentration in the roots to that in soil [41,42,43]. According to MacFarlane et al. (2007) [44], TF is defined as the ratio of the concentration of metals in the leaves compared to those in the roots.

BCF values for nine dominant plant species from the two areas are shown in Table 2. The highest BCF root values were registered for Cd in *Scolymus hispanicus* (12.61) and in *Festuca ovina* (10.90) growing in the tailings as well as in *Cleome brachycarpa* (8.35) in the agricultural soil. These plant species are well developed in these areas and can be used for phytostabilization purposes. The content of bioavailable Cd in soil will decrease, thus, soil pollution will decrease and the transfer of Cd to food chains in toxic quantities will be reduced.

It should be noted that the BCF indicates different contents in different areas. In the tailings, a higher content of BCF in Co (2.82 mg/kg), Cr (1.17 mg/kg), and Zn (1.31 mg/kg) was found in the *Festuca ovina*, and a higher content of Cd was found in *Carlina involucrata* (1.45 mg/kg) and *Cleome brachycarpa* (1.49 mg/kg). In the agricultural soil, *Peganum harmala* had a higher content of Cd (4.70 mg/kg) and Cr (1.3 mg/kg). This can be explained by the differences in the soil concentrations of the metals in these two areas, as well as by the physiological activities of some plant species, which can have a direct impact on metal mobilization and thus on the concentrations of metals in plant tissues. All these plants had very low TF values and can be used in phytostabilization.

These results are in agreement with the study by El Berkaoui et al. (2021) [26] who studied the phytostabilization of phosphate. Similar to the results of the present study, they found the *Festuca ovina* species to be suitable for phytostabilization of phosphates. Moreover, this study agrees with that of Wu et al. (2021) [17] on the phytoremediation of a pyrite mine in China. Like the results of the present study, they found also Asteraceae suitable for the phytostabilization of the mine tailings (*Chrysanthemum indicum, Conyza canadensis, Senecio scandens*).

The TF values were different in the two areas. In the tailings, the higher TF values (>1) were recorded in *Salvia aegyptiaca* for As (1.06), Cr (1.06), and Zn (1.47); in *Colocynthis vulgaris* for As (4.81), Cd (14.06), Cr (1.91), Cu (1.84), and Pb (3.08); in *Paganum harmala* for Cd (8.11), Cr (2.25), Cu (6.31), Ni (1.03) and Pb (3.37). The higher TF values in the agricultural soil were detected in *Festuca ovina* for As (1.15), Cr (1.06), Cu (1.41), and Pb (1.27); in *Colocynthis vulgaris* for As (6.20), Co (1.34), Cr (1.94), Ni (1.24), and Pb (6.10); in *Cleome brachycarpa* for Cu (1.62); in *Stipa retorta* for Cr (1.67), and Ni (1.25).

This indicates that these plants transferred metals to their shoots. All these plants had low BCF values and can be suitable for phytoextraction purposes.

## 4. Conclusions

This article aimed to determine the potential of the abandoned Kettara mine area plant species for phytoremediation of contaminated soils. The study showed that the mine tailings are contaminated with Cu and Pb while the agricultural soils were contaminated with As and Cu. A significant correlation was found between total soil and bioavailable Pb metal fraction, whereas a low correlation was found for As and Cu. The metal contents of nine examined species harvested in two areas revealed that the plant species differ in their capacity to accumulate metals. Shoots in most of plant species accumulate less metal than their roots, which shows the plant’s ability to absorb/fix these metals by the roots but then to limit their mobility in their interior. This indicates the capability of plants to balance the translocation and uptake of metals under polluted conditions.

*Scolymus hispanicus*, *Festuca ovina*, *Cleome brachycarpa*, *Carlina involucrata*, and *Peganum harmala* showed higher values of BCF than the other four dominant plant species. In the above-mentioned plant species, the TF values are low, showing their phytostabilization ability. Indeed, these species are suitable for use in phytoremediation of the Kettara mine area. The species selection is based on metal accumulation in roots and shoots of plants, and detailed studies are needed to investigate the phytoremediation potential of these species in both pot culture and field research. These species could also be good models for the phytostabilization of phosphate waste and, therefore, the completion of the last phase of the Kettara mine rehabilitation project.

## Figures and Tables

**Table 1:** Total concentrations and mobile and mobilizable fractions of metals and metalloids in the rhizospheric soils (mean ± SD, for three repetitions) in mg/kg and pH.

	Areas	As	Cd	Co	Cr	Cu	Ni	Pb	Zn	pH
Total	Tailings	17.57 ± 2.07 ^ab^	0.25 ± 0.05 ^b^	8.41 ± 4.21 ^b^	59.78 ± 17.73 ^c^	177.64 ± 28.52 ^b^	24.48 ± 3.44 ^b^	116.80 ± 28.72 ^c^	150.99 ± 49.22 ^b^	6.87
	Agricultural soil	25.07 ± 18.20 ^b^	0.32 ± 0.12 ^b^	8.81 ± 4.06 ^b^	42.74 ± 4.78 ^b^	251.96 ± 161.76 ^b^	31.05 ± 4.44 ^c^	94.48 ± 3.48 ^b^	115.97 ± 8.49 ^b^	7.34
Mobilizable EDTA	Tailings	0.15 ± 0.04 ^a^	0.02 ^a^	0.08 ± 0.05 ^a^	-	7.37 ± 1.93 ^a^	-	4.64 ± 3.53 ^a^	2.89 ± 1.82 ^a^	
	Agricultural soil	0.26 ± 0.08 ^a^	0.02 ^a^	0.53 ± 0.45 ^a^	-	4.72 ± 3.43 ^a^	-	1.75 ± 0.90 ^a^	2.07 ± 0.94 ^a^	
Mobile CaCl_2_	Tailings	0.05 ^a^	0.02 ^a^	0.05 ^a^	-	0.02 ^a^	-	0.08 ± 0.04 ^a^	0.05 ± 0.04 ^a^	
	Agricultural soil	0.05 ^a^	0.02 ^a^	0.05 ^a^	-	0.02 ^a^	-	0.07 ± 0.02 ^a^	0.01 ^a^	

The same letter within each column indicates no significant difference according to the Student–Newman–Keuls test at 95% confidence limit (*p* ≤ 0.05).

**Table 2:** Bioconcentration factor (BCF) and translocation factor (TF) of trace elements in the sampled species from the tailings and the agricultural soil.

		BCF								TF							
Areas	Species	As	Cd	Co	Cr	Cu	Ni	Pb	Zn	As	Cd	Co	Cr	Cu	Ni	Pb	Zn
**Tailings**	*Festuca ovina*	0.19	10.90	2.82	1.17	0.97	0.51	0.14	1.31	0.56	0.49	0.02	0.28	0.19	0.36	0.50	0.31
	*Carlina involucrata*	0.05	1.45	0.30	0.15	0.60	0.28	0.02	0.19	0.85	0.85	0.74	2.27	0.69	1.65	3.75	3.87
	*Salvia aegyptiaca*	0.04	1.23	0.35	0.19	0.28	0.25	0.05	0.16	1.06	1.06	0.28	1.06	0.21	0.92	0.84	1.47
	*Colocynthis vulgaris*	0.03	0.85	0.06	0.27	0.11	0.35	0.03	0.51	4.81	14.06	0.96	1.91	1.84	0.98	3.08	0.69
	*Scolymus hispanicus*	0.08	12.61	0.10	0.14	0.53	0.34	0.01	0.37	0.44	0.45	0.63	0.54	0.20	0.43	1.76	0.94
	*Peganum harmala*	0.03	0.86	0.07	0.07	0.01	0.16	0.01	nd	0.96	8.11	0.96	2.25	6.31	1.03	3.37	nd
	*Cleome brachycarpa*	0.05	1.49	0.11	0.21	0.05	0.22	0.05	0.39	0.84	0.84	0.84	0.83	0.62	0.78	0.95	0.37
**Agricultural soil**	*Festuca ovina*	0.03	0.89	0.08	0.49	0.03	0.25	0.11	0.25	1.15	0.92	0.92	1.06	1.41	0.93	1.27	0.88
	*Colocynthis vulgaris*	0.02	4.12	0.06	0.27	0.04	0.22	0.03	0.47	6.20	2.70	1.34	1.94	0.02	1.24	6.10	0.64
	*Glaucium corniculata*	0.03	0.98	0.09	0.28	0.01	0.16	0.09	0.07	0.94	0.94	0.94	0.69	0.53	0.76	0.46	0.50
	*Peganum harmala*	0.04	4.70	0.08	1.53	0.08	1.07	0.05	0.81	0.49	0.14	0.73	0.05	0.59	0.08	0.27	0.40
	*Cleome brachycarpa*	0.07	8.35	0.06	0.25	0.06	0.20	0.21	0.48	0.58	1.33	0.98	0.50	1.62	0.57	0.45	0.72
	*Stipa retorta*	0.03	0.97	0.09	0.16	0.13	0.13	0.05	0.18	0.82	0.82	0.82	1.67	0.19	1.25	0.71	0.68

## Data Availability

Not applicable.
